# Plasma Biomarkers of the Glycocalyx in Delayed Cerebral Ischemia After Aneurysmal Subarachnoid Hemorrhage

**DOI:** 10.3390/brainsci16080840

**Published:** 2026-08-07

**Authors:** Karmen Aslankurt, Hanna Schenck, Sander M. J. van Kuijk, Govert Hoogland, Inger de Ridder, Onno Teernstra, Jim Dings, Marcel Aries, Michael Veldeman, Hans Vink, Yasin Temel, Rick van Lanen, Roel Haeren

**Affiliations:** 1 Mental Health and Neuroscience Research Institute, Maastricht University, 6200 MD Maastricht, The Netherlands; karmen.aslankurt@maastrichtuniversity.nl (K.A.); hanna.schenck@mumc.nl (H.S.); g.hoogland@maastrichtuniversity.nl (G.H.); i.de.ridder@mumc.nl (I.d.R.); o.teernstra@mumc.nl (O.T.); jim.dings@mumc.nl (J.D.); marcel.aries@mumc.nl (M.A.); y.temel@mumc.nl (Y.T.); rick.van.lanen@mumc.nl (R.v.L.); 2 Department of Neurosurgery, Maastricht University Medical Center+, P. Debyelaan 25, 6202 AZ Maastricht, The Netherlands; 3 Department of Clinical Epidemiology and Medical Technology Assessment, Maastricht University Medical Center+, 6202 AZ Maastricht, The Netherlands; sander.van.kuijk@mumc.nl; 4 Department of Neurology, Maastricht University Medical Center+, 6202 AZ Maastricht, The Netherlands; 5 Department of Intensive Care, Maastricht University Medical Center+, 6202 AZ Maastricht, The Netherlands; 6 Department of Neurosurgery, RWTH Uniklinikum Aachen, 52074 Aachen, Germany; mveldeman@ukaachen.de; 7 GlycoCalyx Research Institute, Alpine, UT 84004, USA; hans.vink@glycocheck.com

**Keywords:** aneurysmal subarachnoid hemorrhage, delayed cerebral ischemia, glycocalyx, biomarker, syndecan-1, hyaluronan

## Abstract

**Highlights:**

**What are the main findings?**

- Plasma syndecan-1 concentrations increase during the first two weeks following aSAH ictus.
- This increase appears to be more prominent in patients with DCI and occurs mainly at DCI onset
- Plasma hyaluronan concentrations are elevated in the early stage of aSAH relative to values reported in the literature

**What are the implications of the main findings?**

- Glycocalyx breakdown might be involved in the pathophysiology of DCI

**Abstract:**

Background: An aneurysmal subarachnoid hemorrhage (aSAH) is a life-threatening neurovascular emergency. Among survivors, the most dreaded complication is delayed cerebral ischemia (DCI), affecting approximately one third of aSAH patients. Several mechanisms contributing to DCI have been described, yet the pathophysiology remains unclear. Disruption of the endothelial glycocalyx plays a key role in these pathomechanisms and is therefore of particular interest. This study aimed to explore changes in plasma glycocalyx breakdown products following aSAH and in relation to the development of DCI. Methods: In this prospective study, plasma samples were collected during a two-week follow-up period after aSAH diagnosis. Baseline plasma samples were collected within 72 h after ictus, followed by two additional samples in the first week and three samples in the second week. In patients who developed DCI, additional daily samples were obtained. DCI was defined using its clinical definition. Glycocalyx breakdown products syndecan-1 and hyaluronan concentrations were measured using enzyme-linked immunosorbent assays (ELISA) kits. Results: Twenty-nine aSAH patients were included, of whom six developed DCI. Overall, median syndecan-1 concentrations increased significantly from baseline (33.72 ng/mL, IQR 23.29–42.55 ng/mL) to the day 14 measurements (44.83 ng/mL, IQR 32.52–62.68) (*p* = 0.002). This increase was more prominent in the DCI group (baseline: 34.35 ng/mL, IQR 33.77–50.99, final measurement: 52.31 ng/mL, IQR 44.98–170.62, *p* = 0.031) compared to the non-DCI group (baseline: 33.07 ng/mL, IQR 23.29–42.32, final measurement: 37.97 ng/mL, IQR 32.52–52.14, *p* = 0.028), and mainly occurred at DCI onset. Median baseline hyaluronan concentrations were increased at baseline (148.81 ng/mL, IQR 90.61–178.93) and subsequently decreased (final measurement 115.96 ng/mL, IQR 99.15–140.35, *p* = 0.263). A comparable decrease in hyaluronan levels was noted in the DCI (baseline: 147.82 ng/mL, IQR 80.63–169.63, final measurement: 117.02 ng/mL, IQR 96.73–200.68, *p* = 0.893) and non-DCI group (baseline: 149.81 ng/mL, IQR 109.16–178.92, final measurement: 114.91 ng/mL, IQR 102.26–136.63, *p* = 0.156). Conclusions: The results from this explorative study indicate that aSAH is associated with glycocalyx breakdown. While hyaluronan levels were high in the acute phase and decreased thereafter, syndecan-1 levels increased during the first two weeks following aSAH in DCI patients. For syndecan-1, this change was more pronounced in DCI patients. Our findings suggest an involvement of glycocalyx breakdown in the pathophysiology of DCI. This study requires validation in larger cohorts to strengthen our results, and establishes the potential of glycocalyx breakdown products as biomarkers for DCI.

## 1. Introduction

An aneurysmal subarachnoid hemorrhage (aSAH) accounts for approximately 5% of all strokes and is associated with high morbidity and mortality. It predominantly affects adults between the ages of 40 and 60 years, thereby imposing a significant socioeconomic burden [1]. The most dreadful complication of aSAH is delayed cerebral ischemia (DCI). DCI occurs in approximately 30% of aSAH patients and is a major contributor to mortality and long-term disability [2]. Consequently, DCI is also a major cost driver of in-hospital costs of aSAH [3,4]. Despite advances in critical care management, accurately predicting which patients will develop DCI remains a significant clinical challenge, and no effective treatment options for DCI are available.

**Figure 1:**
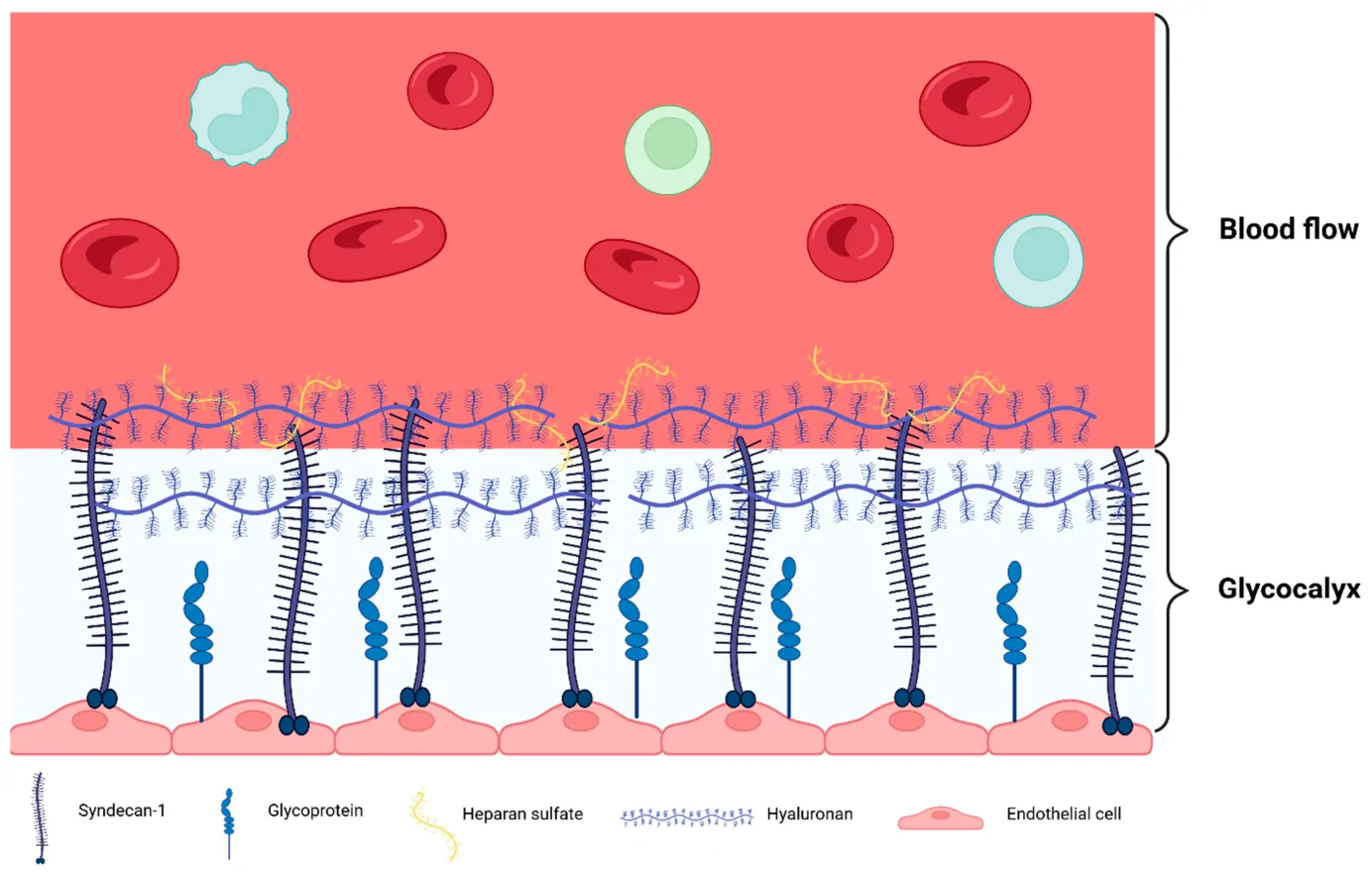
Schematic representation of the endothelial glycocalyx with its associated proteoglycans (e.g., syndecan-1), glycosaminoglycans (e.g., heparan sulfate and hyaluronan) and glycoproteins. Syndecan-1 is strongly attached to endothelial cells via its transmembrane domain. Heparan sulfate is covalently attached to the syndecan-1 core protein. Hyaluronan, a long polymer, weaves through and maintains a loose connection to the glycocalyx, forming a flexible molecular scaffold.

The pathophysiology of DCI is complex and multifactorial. Proposed mechanisms include microvascular dysfunction, microthrombosis, neuroinflammation, loss of cerebral autoregulation, cortical spreading depolarization and blood-brain barrier dysfunction, among others [5,6]. The endothelial glycocalyx, a carbohydrate-rich layer lining the luminal surface of blood vessels, plays a key role in the regulation of the underlying microvascular processes [7]. The glycocalyx is primarily composed of membrane-bound proteoglycans, such as syndecan-1, to which one or more glycosaminoglycans (GAG) side chains, including heparan sulfate and hyaluronan, are attached [8,9]. Syndecan-1 is one of the principal structural proteoglycans of the glycocalyx, and is strongly anchored to the endothelium due to its transmembrane structure (Figure 1). Hyaluronan is a relatively long GAG, accounting for 50–90% of GAGs and more loosely connected within the luminal part of the glycocalyx, making it more exposed to the blood flow. The glycocalyx contributes to vascular permeability control, shear stress sensing, mechanotransduction, and attenuation of leukocyte and platelet adhesion [5]. Disruption of the glycocalyx has been implicated in various pathological conditions, such as sepsis and ischemia-reperfusion injury and potentially also in the development of DCI [5,10,11]. Two previous studies indeed described increased plasma concentrations of syndecan-1 and hyaluronan in aSAH patients, and specifically in relation to cerebral vasospasm [10,12]. While these findings are encouraging, both studies were limited by very small sample sizes. (*N* = 3, and *N* = 18).

Therefore, the aim of the current study was to prospectively characterize temporal changes of plasma syndecan-1 and hyaluronan concentrations in 30 aSAH patients during the first two weeks following aSAH ictus. Furthermore, we aimed to compare temporal patterns of syndecan-1 and hyaluronan shedding between patients who developed DCI and those who did not, and assess whether these glycocalyx components could form potential biomarkers for DCI.

## 2. Methods

### 2.1. Ethical Considerations

The protocol was approved by the Medical Ethics Review Committee (METC) of academisch ziekenhuis Maastricht/Universiteit Maastricht (approval number NL76189.068.21). The full study protocol was registered at ClinicalTrials.gov (NCT05483751) and published recently [13]. Written informed consent was obtained from each patient or the patient’s legal representative if the patient was mentally incapacitated and unable to give informed consent. Delayed informed consent was obtained from mentally incapacitated patients if clinical improvement occurred.

### 2.2. Study Population

In this single-center prospective study, patients were included if they were 18 years of age or older and diagnosed with aSAH confirmed by computed tomography (CT) in combination with CT-angiography (CTA) and/or digital subtraction angiography (DSA). Inclusion within 72 h after symptom onset (ictus) was required to enable a baseline assessment before the occurrence of DCI. Patients were excluded in cases of expected imminent death within 24 h, non-aneurysmal SAH, or inability to communicate in Dutch, English or French.

Since this study is part of the larger PDMMS project (NCT05483751), patients who suffered from ophthalmic or oral infections were also excluded due to the potential interference with our microcirculatory measurements on the eye and under the tongue. Feasibility of the full set of study measurements has been published previously [14].

### 2.3. Study Procedures

Baseline blood samples were taken within 72 h after ictus (baseline). Two additional blood samples were taken within a week after patient inclusion with an interval of one or two days. In the second week, three additional blood samples were taken with an interval of one or two days, yielding a total of six blood samples. In patients who developed DCI, additional daily blood samples were collected during the DCI episode. DCI was defined according to established consensus clinical diagnosis, i.e., the occurrence of a new neurological deficit or decrease in the Glasgow Coma Scale (GCS) of at least two points lasting for at least one hour, after the exclusion of other causes [15]. The neurological condition of the patients was assessed by the treating physician and nursing team. The end of DCI was defined as the moment the treating physician noted a resolution of neurological symptoms. Blood samples were collected early in the morning when patients were fasting to reduce potential effects of blood glucose concentrations on glycocalyx integrity.

For this article, we included a baseline (acute phase) measurement, the measurement at DCI occurrence, the latest available measurement before DCI was diagnosed, the measurement after DCI resolved, and the final measurement (14 days after ictus). For each sample, five milliliters of venous blood were collected in ethylenediaminetetraacetic acid (EDTA) collection tubes. To obtain plasma, blood samples were centrifuged in a horizontal rotor for fifteen minutes at 1200× *g* at 20 degrees Celsius (°C) within four hours after retrieval. Plasma was collected in aliquots and stored in a −80 °C freezer until further analysis to determine plasma concentrations of glycocalyx-related breakdown products.

### 2.4. Glycocalyx-Related Breakdown Products Assessment in Human Plasma

In this study, we opted to include syndecan-1 and hyaluronan as glycocalyx components of interest. Both components have been studied in relation to aSAH previously [10,12] and their assessment tools are widely available. Besides these similarities, both components are of particular interest as they strongly differ in their location and role within the glycocalyx. Firstly, the location of both components is different. While syndecan-1 forms an anchor molecule for the gel-like structure to the endothelium, hyaluronan is more loosely bound within the glycocalyx matrix. Secondly, these components have different functions within the glycocalyx. For both glycocalyx breakdown products, enzyme-linked immunosorbent assays (ELISAs) were performed according to the manufacturer’s instructions to determine their plasma concentrations: syndecan-1 (Human CD138 ELISA Kit, Catalog No. 950.640, Medix Biochemica, Espoo, Finland) and hyaluronan (Hyaluronan Enzyme-Linked Immunosorbent Assay, Catalog No. K-1200, Echelon Biosciences, Salt Lake City, UT, USA). The assays were carried out in the same laboratory under standard conditions. The CLARIOstar^®^ (BMG LABTECH, Ortenberg, Germany), which is a multi-mode plate reader, was used to measure the absorbance of the color complexes and compute syndecan-1 and hyaluronan concentrations according to the corresponding standard curve. Plasma samples were measured in triplicate. Assay performance was verified using plasma samples from healthy controls prior to analysis of patient samples.

### 2.5. Patient Data

Data collection included general patient demographic characteristics, including sex, age, medical history, medication and smoking status. Clinical and radiological presentation at admission was documented according to the World Federation of Neurological Surgeons (WFNS) grading scale and the modified Fisher grading scale (mFisher), respectively. The WFNS score is based on the GCS and the presence of focal neurological deficits, ranging from Grade I to Grade V, with Grade V indicating the most severe aSAH grade [16]. The mFisher grades the severity of aSAH based on CT and the associated risk of vasospasm, ranging from grade 1 to grade 4, with grade 4 indicating a high risk for vasospasm [17]. At each measurement, the clinical condition of the patient was assessed using the GCS to determine the level of consciousness.

### 2.6. Statistical Analysis

Statistical analysis was performed with IBM SPSS Statistics version 29.0.1. The assumption of normality was checked with Q-Q Plots and the Shapiro–Wilk test. Baseline patient demographics were summarized as the mean and standard deviation (SD) or as a count and percentage, and stratified by DCI and non-DCI. Between–group differences were tested using the independent samples *t*-test or Mann–Whitney U test for continuous parameters, and Fisher’s exact test for categorical variables.

The Wilcoxon Signed-Rank Test was used to compare syndecan-1 and hyaluronan concentrations between the baseline (the first measurement within 72 h after ictus) and the final measurement (14 days after ictus) for all aSAH patients, for the DCI group and for the non-DCI group. Subsequently, we compared syndecan-1 and hyaluronan concentrations before DCI occurrence and during DCI, as well as during DCI and the first measurement after DCI ended. Given the exploratory nature of the study, no correction for multiple testing was applied. A *p* < 0.05 was considered statistically significant.

## 3. Results

### 3.1. Patient Characteristics

A total of 88 aSAH patients were screened, of whom 38 met the eligibility criteria. In eight cases, participation was declined, resulting in 30 included participants. For the final analysis of demographics and syndecan-1 and hyaluronan levels, one patient was excluded because of ischemic lesions on baseline imaging, which precluded a valid baseline before DCI occurrence. Of the remaining 29 patients, 24 (82.8%) patients were female and the median age was 58 years (Table 1). Seven patients had a medical history of hypertension and 18 were smokers. The majority of the aSAH patients had a low WFNS score (WFNS 1 or 2) (*N* = 21, 72.4%), and a mFisher score of 3 (*N* = 13, 44.8%) was most common. Mortality at 6 months was 14% (*N* = 4).

A total of 6 patients developed DCI during the first two weeks following aSAH. Patients who developed DCI were younger than those who did not (52 ± 7.3 versus 59 ± 11.4 years of age). In the DCI subgroup, all patients had a mFisher score of 3 or 4 and the mortality rate was higher than in the non-DCI subgroup. (Table 1).

### 3.2. Syndecan-1

**Figure 2:**
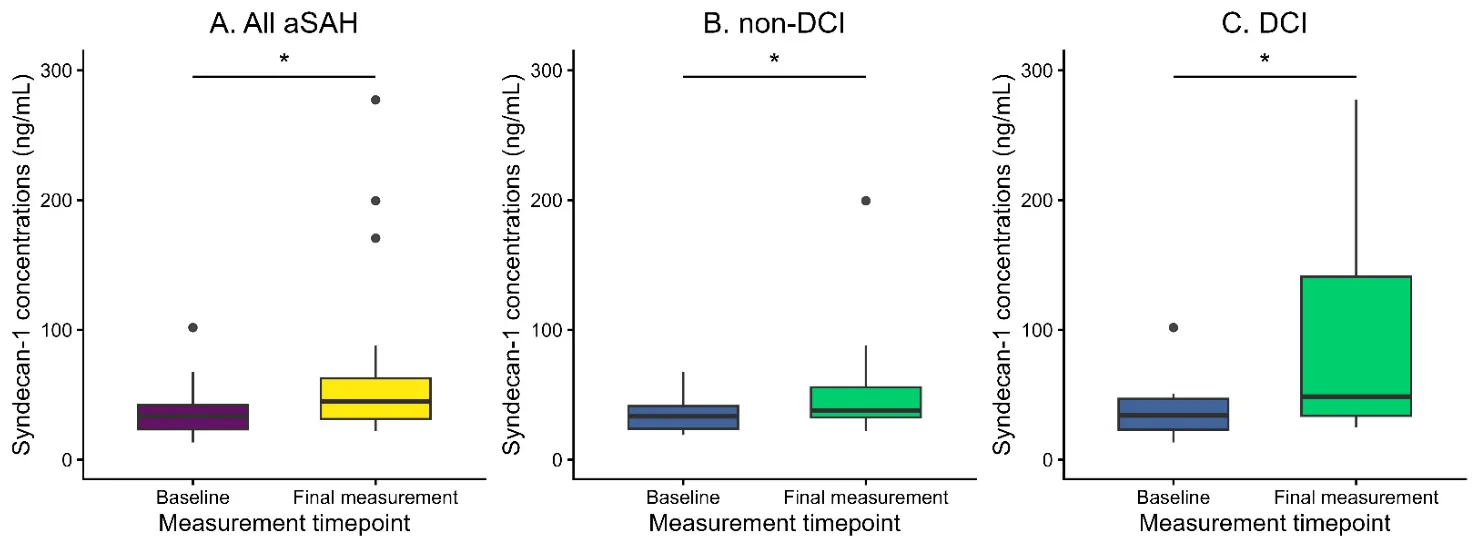
Syndecan-1 concentrations in all aSAH patients (**A**), in non-DCI patients (**B**) and DCI patients (**C**) at baseline and final measurement. * Indicates a statistically significant difference between baseline and the final measurement (*p* < 0.05). One patient was excluded from baseline analysis since results fell outside of the detectable range of the ELISA standard curve. Hence, *N* = 28 for all aSAH patients, *N* = 22 for patients without DCI and *N* = 6 for patients with DCI. For the final measurement, data from 8 patients were excluded from analysis. Two patients passed away before the final measurement, four patients did not undergo blood collection, and two patients had syndecan-1 values which fell outside the detectable range of the ELISA standard curve. Hence, for the final analysis we included *N* = 21 for all aSAH patients, *N* = 17 for patients without DCI and *N* = 4 for patients with DCI. Patients who died before the final measurement had baseline syndecan-1 values comparable to the overall cohort (25.42 ng/mL (range 23.08–27.76)).

For all aSAH patients, we noted that median syndecan-1 concentrations increased significantly from 33.72 ng/mL (IQR 23.29–42.55) to 44.83 ng/mL (IQR 32.52–62.68) (*p* = 0.002). Baseline syndecan-1 concentrations of patients with DCI and without DCI were comparable (*p* = 0.955). In non-DCI patients, syndecan-1 increased from 33.07 ng/mL (IQR 23.29–42.32) to 37.97 ng/mL (IQR 32.52–52.14) (*p* = 0.028), whereas syndecan-1 increased from 34.35 ng/mL (IQR 33.77–50.99) to 52.32 ng/mL (IQR 44.98–170.62) (*p* = 0.031) in DCI patients (Figure 2). Although the magnitude of the increase was greater for patients with DCI, this difference was non-significant (*p* = 0.059) due to large between-patient variability.

### 3.3. Hyaluronan

**Figure 3:**
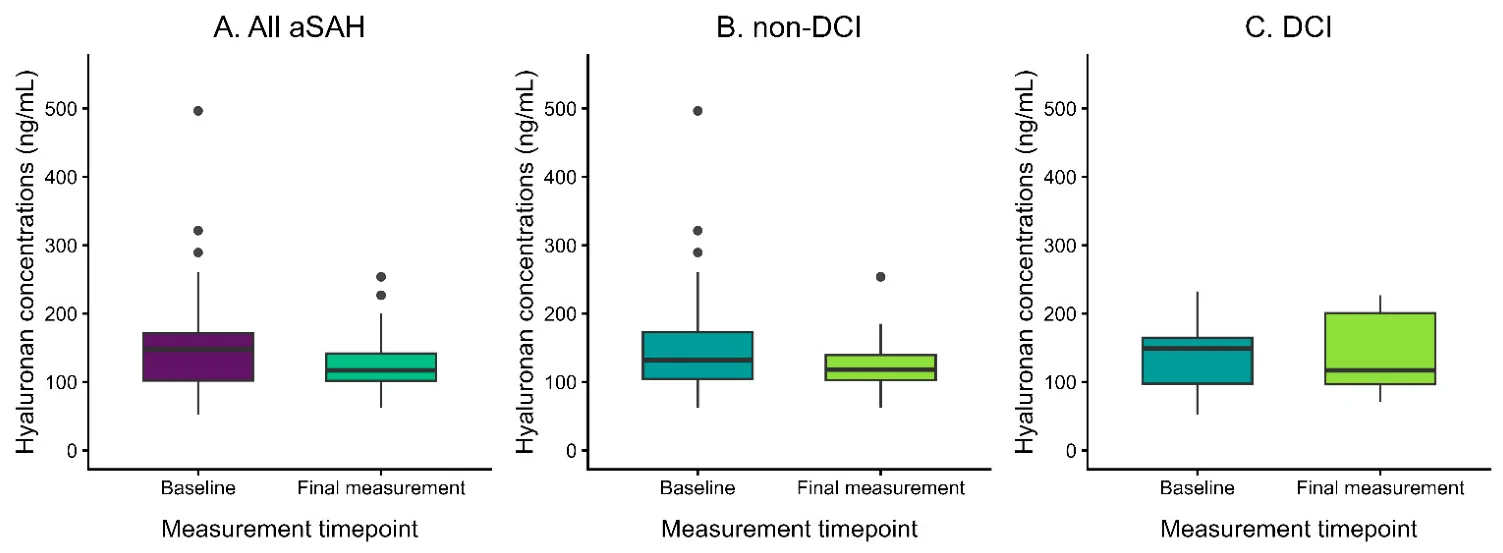
Hyaluronan concentrations in all aSAH patients (**A**), in non-DCI patients (**B**) and DCI patients (**C**) at baseline and final measurement. Baseline data from two patients were excluded since they fell outside the detectable range of the ELISA standard curve. For the baseline measurement analysis, we included 27 aSAH patients, 21 non-DCI patients, and 6 DCI patients. For the final measurement, data from 9 patients were excluded from final analysis: two patients passed away before the measurement, three patients did not undergo blood collection, and four patients had hyaluronan concentrations outside of the ELISA standard curve. Hence, for the final measurement analysis, we included 20 aSAH patients, 16 non-DCI patients and 4 DCI patients. Patients who died before the final measurement had baseline hyaluronan values comparable to the overall cohort (214.11 ng/mL (range 167.4–260.82)).

For hyaluronan concentrations in all aSAH patients, a decrease was noted between baseline and final measurement from 148.81 ng/mL (IQR 90.61–178.93) to 115.96 ng/mL (IQR 99.15–140.35), albeit non-significant (*p* = 0.263). Hyaluronan baseline concentrations (*p* = 0.077) as well as hyaluronan changes (*p* = 0.359) were comparable between non-DCI patients (baseline: 149.81 ng/mL (IQR 109.16–178.92); final: 114.91 ng/mL (IQR 102.26–136.63)) and DCI patients (baseline: 147.82 ng/mL (IQR 80.63–169.63); final: 117.02 ng/mL (IQR 96.73–200.68) (Figure 3).

### 3.4. Impact of DCI on Syndecan-1 and Hyaluronan Concentrations

In patients who developed DCI, we estimated the effect of DCI onset on syndecan-1 and hyaluronan concentrations by comparing syndecan-1 and hyaluronan concentrations between latest measurement before DCI onset (pre-DCI) and the measurement directly following DCI onset (DCI). The DCI measurement was always performed within 24 h after DCI onset with a median time from DCI onset of 1 day (IQR 1-1).

**Figure 4:**
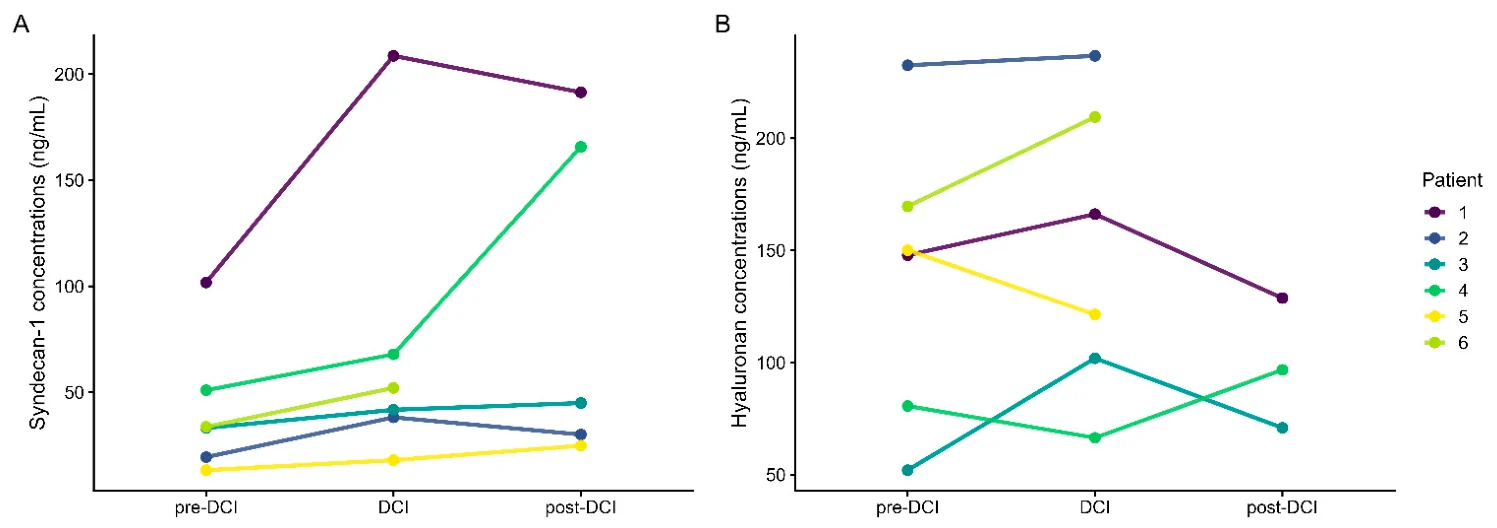
Syndecan-1 (**A**) and hyaluronan concentrations (**B**) pre-DCI, DCI and post-DCI. For syndecan-1, *N* = 6 for pre-DCI and DCI, and *N* = 5 for post-DCI. For hyaluronan, *N* = 6 for pre-DCI and DCI, and *N* = 3 for post-DCI. Identical colors were assigned to each participant across (**A**,**B**).

For syndecan-1, we were able to perform this analysis in six patients and noted a significant increase from 33.49 ng/mL (IQR 19.45–50.99) to 46.95 ng/mL (IQR 38.24–67.94) (*p* = 0.028) directly following the onset of DCI. All patients showed an increase in syndecan-1 concentrations from pre-DCI to DCI. Three patients showed an increase from DCI to post-DCI, whereas two patients showed a decrease. Hyaluronan concentrations were also assessed in six DCI patients, and we found that hyaluronan concentrations remained stable following DCI onset. Specifically, four patients showed an increase in syndecan-1 concentrations between pre-DCI and DCI, whereas two patients showed a decrease (Figure 4).

At the end of DCI (post-DCI), syndecan-1 concentrations (*n* = 5, one DCI patient was excluded because concentrations fell outside the detectable range of the ELISA standard curve) remained stable (DCI: 46.95 (IQR 38.24–67.94), post-DCI: 44.98 (IQR 30.12–165.71) (*p* = 0.893). Three patients showed an increase from DCI to post-DCI, whereas two patients showed a decrease. Hyaluronan concentrations (*n* = 3, three patients were excluded because concentrations fell outside the detectable range of the ELISA standard curve) showed a notable yet non-significant decrease (DCI: 143.80 ng/mL (IQR 236.74–66.43), post-DCI: 96.73 ng/mL (IQR 70.91–128.75) (*p* = 0.285). Two patients showed a decrease between pre-DCI to post-DCI, whereas one patient showed an increase (Figure 4).

## 4. Discussion

To our knowledge, this is the first prospective study to investigate both syndecan-1 and hyaluronan concentrations in a larger group of aSAH patients. We found that syndecan-1 increased significantly during the first two weeks following aSAH ictus. Although not statistically significant, syndecan-1 levels appeared to increase more markedly in patients who developed DCI, and appeared to be primarily driven by a steeper increase observed at DCI onset. Simultaneously, hyaluronan concentrations gradually decreased in both the DCI and non-DCI groups.

### 4.1. Syndecan-1 in aSAH and DCI

The baseline measurement of syndecan-1 concentrations was performed within 72 h after aSAH ictus. At that time point, we found a median syndecan-1 concentration of 33.72 ng/mL (IQR 23.29–42.55) for all aSAH patients. Astapenko et al. analyzed and pooled mean and median concentrations of syndecan-1 from studies with healthy controls, and calculated a median of 17 ng/mL as the hypothetic median value for syndecan-1 in healthy controls [18]. However, due to variability among ELISA assays, these values may vary. This may explain the variation in syndecan-1 concentrations observed in previous studies in aSAH patients. Grybel-Brask et al. reported median syndecan-1 plasma concentrations of around 10–11 ng/mL in 30 aSAH patients measured on day 3 after aSAH ictus [19]. Another study by Schick et al. found median plasma syndecan-1 concentrations of 23 ng/mL (IQR 17–36) in 18 patients during the early phase of the aSAH [12]. Bell et al. also evaluated syndecan-1 plasma concentrations in three aSAH patients, and described concentrations ranging from 12 to 50 ng/mL [10]. Using multiplex immunoassays, Serlé et al. reported early syndecan-1 levels of 2.2 ng/mL (1.8–2.7), which in turn were lower than the syndecan-1 levels of the included healthy controls in this study [20]. It must be noted that these healthy controls had a high rate of diseases that are well known to reduce glycocalyx integrity, such as diabetes mellitus and hypertension, which may have affected these results.

In the course of the aSAH, we noted an increase of around 11 ng/mL in syndecan-1 concentrations in all aSAH patients. This is in line with the findings from Gybel-Brask et al. and Serlé et al., reporting on an increase of median syndecan-1 plasma concentrations in the first 2 weeks following aSAH ictus [19,20]. The increase in our cohort was most prominent in patients who developed DCI. While Gybel-Brask et al. did not differentiate DCI from non-DCI patients in their aSAH cohort, Bell et al. included only patients who developed DCI and noted a clear increase in syndecan-1 concentrations in all three DCI patients [10,19]. In their series of 18 patients, Schick et al. reported cerebral vasospasms in 10 patients [12], and noted an increase in syndecan-1 concentrations around day 6–7 in these patients. Notably, an increase in smaller magnitude was observed in patients who did not develop vasospasms [12]. However, cerebral vasospasms are not interchangeable for the diagnosis of DCI, as explained, making these results less comparable. In contrast to our findings, Serlé et al. found no significant difference in syndecan-1 levels between DCI and non-DCI patients [20]. Overall, syndecan-1 levels increased during the first two weeks of aSAH ictus in our study, which is in line with previous studies; however, whether this increase is significantly more pronounced in DCI patients than non-DCI patients requires further investigation.

In our cohorts, we found increased syndecan-1 levels early after aSAH ictus, with a secondary increase in the two weeks thereafter. Physiologically, this early increase may be a direct result of the hemorrhage and its early sequelae. Specifically, an aSAH causes a sharp rise in intracranial pressure leading to transient global ischemia activating several inflammatory pathways in response to damage-associated molecular patterns (DAMPs) [21,22,23]. Inflammatory markers, such as interleukin-1 and interleukin-6, rapidly increase and there is a massive infiltration of neutrophils and monocytes at the site of injury, resulting in microglia activation and formation of reactive oxygen species, further exacerbating the inflammatory response [22,23,24]. In the 3 to 14 days after this so-called early brain injury phase, blood-brain barrier disruption, neurovascular uncoupling, neuroinflammation, cortical spreading depolarization and neuronal necrosis occur [5,25,26,27]. Matrix metalloproteinases (MMPs), such as MMP-9, get upregulated in response to inflammation, and are a major source of cleaving syndecan-1 from the glycocalyx, causing enhanced concentrations in plasma [28]. In addition to these processes following aSAH, aSAH patients frequently develop complications, such as pulmonary edema, cardiomyopathy, and infections during hospitalization which can themselves cause glycocalyx degradation [29,30,31], further contributing to syndecan-1 shedding in the first 14 days after aSAH ictus. These processes may explain the increase in syndecan-1 concentrations in our aSAH cohort.

Interestingly, although the difference was not statistically significant, the magnitude of increase in syndecan-1 appeared to be greater in patients who developed DCI than in those who did not, with a steeper increase in syndecan-1 appearing to occur at DCI onset. This may suggest an association between syndecan-1 and DCI and indicates that syndecan-1 warrants further investigation as a possible biomarker for DCI. However, whether glycocalyx injury precedes the development of DCI or whether DCI itself induces glycocalyx breakdown cannot be determined based on our study design and the limited number of DCI patients.

### 4.2. Hyaluronan in aSAH and DCI

Hyaluronan concentrations decreased gradually in the first two weeks after aSAH ictus. This decrease was comparable between patients who developed DCI and those who did not. Similar to syndecan-1, a healthy range for hyaluronan has not been established yet, but in the healthy population plasma concentrations of hyaluronan are typically below 100 ng/mL [32]. Hence, hyaluronan concentrations in our cohort (median 148.81 ng/mL) reflect increased and potentially pathological concentrations, especially at baseline. Schick et al. also observed increased plasma hyaluronan concentrations in patients with aSAH compared to healthy controls, 131 ng/mL and 92 ng/mL, respectively. In patients who developed cerebral vasospasm, hyaluronan concentrations further increased and reached a median of 206 ng/mL by day 7 after aSAH, which suggests ongoing hyaluronan breakdown. This contrasts our results strongly with a decrease in hyaluronan in all aSAH patients, regardless of the development of DCI. Again, Schick et al. used vasospasm as a surrogate marker, limiting direct comparability [12].

The higher hyaluronan concentrations at baseline compared to concentrations reported in the literature suggest acute degradation of hyaluronan in response to aneurysm rupture and early brain injury, as described earlier. As mentioned, the early brain injury phase is characterized by a sharp increase in inflammatory cytokines. These may activate hyaluronidases (HYALs), such as HYAL1 and HYAL2, which cleave hyaluronan into smaller low-molecular weight fragments that are pro-inflammatory and serve as DAMPs [33,34]. Hence, the acute phase following aSAH could be particularly characterized by hyaluronan release from the glycocalyx. Hyaluronan concentrations returning to near physiological concentrations after 14 days may either suggest that hyaluronan release from the glycocalyx normalized again or the biosynthesis of hyaluronan itself is impaired due to prolonged inflammation [35]. In fact, oscillatory and disturbed blood flow causes decreased activity of transcription factor Krüppel-like Factor 2 (KLF2), which has been associated with reduced expression of hyaluronan synthase, the major enzyme responsible for hyaluronan biosynthesis in endothelial cells [36,37]. The fact that there is no clear difference in hyaluronan concentrations between our patients who develop DCI and those who did not may suggest that hyaluronan is associated with acute damage after aSAH rather than secondary processes that lead to the development of DCI.

### 4.3. Distinct Patterns in Syndecan-1 and Hyaluronan Concentrations

In our cohort, syndecan-1 and hyaluronan concentrations exhibit distinct patterns during the course of aSAH. These distinct findings may in fact reflect the distinct phases as well as the severity of glycocalyx injury in relation to the different pathological incidents and phases related to aSAH and DCI. For example, the cerebral conditions directly following aSAH ictus are characterized by acute inflammation. In this acute phase, we noted increased plasma concentrations of hyaluronan in our aSAH patients compared to the values of healthy controls reported in the literature. Hyaluronan is supposedly more sensitive to acute glycocalyx injury, as was previously suggested in patients with advanced stage of cardiac failure [38]. This suggestion was later confirmed in another study demonstrating that hyaluronan concentrations were significantly elevated as early as 60 min after multiple organ failure [39]. The acute release of hyaluronan may physiologically be explained by its location within the glycocalyx. Hyaluronan is mostly situated in the luminal part of the glycocalyx (Figure 1), and is non-covalently bound to endothelial cells via the CD44 receptor or attached to hyaluronan synthases [8,40]. Hence, hyaluronan has no direct attachment to the endothelial cell membrane, and can thus be released more easily in response to inflammation and vascular injury.

In contrast to hyaluronan, we found that syndecan-1 levels were only slightly increased in the acute phase followed by a secondary increase in the two weeks thereafter. This is in line with the study on patients with multiple organ failure describing a more delayed release of syndecan-1 compared to hyaluronan [39]. Moreover, syndecan-1 release in patients with cardiac failure was mostly associated with chronic injury rather than acute injury [38]. Syndecan-1 is directly attached to the endothelial cell membrane via its transmembrane domain [8]. As such, the release of syndecan-1 may reflect more chronic and severe glycocalyx disintegration and endothelial cell injury. The delayed increase of syndecan-1 in our aSAH patients may thus reflect the ongoing pathophysiological processes following early brain injury that are indeed characterized by endothelial cell injury in the phase following early brain injury [5,41]. This suggestion is further supported by the observation that syndecan-1 concentrations appeared to increase more steeply around the time of DCI onset. At a cellular level, DCI is characterized by the development of parenchymal hypoperfusion and ischemia which is the result of neurovascular uncoupling, endothelial cell dysfunction and increased blood-brain barrier permeability [5]. All these findings are associated with severe glycocalyx injury and endothelial dysfunction [42].

Several biomarkers have been investigated for the prediction of DCI. To date, biomarker research in aSAH has primarily focused on inflammatory mediators, coagulation-related pathways, oxidative stress markers and neuronal injury markers. For instance, early increases in blood C-reactive protein (CRP) have been associated with DCI development, as well as an increased neutrophil-to-lymphocyte ratio (NLR) [43,44]. In addition, patients who developed DCI exhibited higher levels of interleukin-6 (IL-6), von Willebrand factor (VWF) and P-selectin, all of which are involved in the inflammation-coagulation cascade [45,46]. Elevated levels of F2-isoprostanes, a marker of oxidative stress, has likewise been associated with DCI development [45]. Furthermore, patients with DCI showed higher concentrations of the blood-brain barrier-associated molecule S100B on day 3, and an early increase in neuroglobin levels [47,48]. Despite recent advancements, no biomarker has yet demonstrated sufficient sensitivity, specificity and reproducibility to support routine clinical practice [45]. This is reflected in the systematic review by Jabbarli et al., which evaluated 257 blood and CSF parameters across 16.914 aSAH patients and found that none of the investigated biomarkers reached level I evidence for DCI prediction [49]. Several factors contribute to this lack of biomarker establishment, including heterogeneity across studies with respect to patient characteristics, DCI definitions, timing of biomarker sampling and laboratory assays used [45]. Moreover, the predominance of small, single-center studies has limited the generalizability and clinical translation of biomarker findings [45,49]. Furthermore, the multifactorial and complex pathophysiology of DCI, which involves various processes, including neuroinflammation, endothelial dysfunction, microthrombosis and blood-brain barrier disruption, complicates the identification of biomarkers assigned to a single mechanistic category [45]. In this regard, glycocalyx degradation markers are of particular interest, since the glycocalyx is implicated in the aforementioned processes [5]. As such, syndecan-1 and hyaluronan may represent a more integrative assessment of DCI pathophysiology as a whole instead of a single process. Clearly, further prospective research is required to determine their diagnostic and prognostic value for DCI prediction.

### 4.4. Strengths and Limitations

This study has several limitations. Firstly, the present study was designed as a single-center study restricting generalizability of our results. Secondly, the patient cohort is relatively small, particularly the number of patients who developed DCI limiting statistical power and precluding robust multivariable analyses and longitudinal modeling. Consequently, residual confounding and overfitting cannot be excluded. We acknowledge that potential confounders, such as sepsis, pneumonia and systemic inflammation, may influence syndecan-1 and hyaluronan levels; however, these were not systematically assessed in this exploratory study due to the small sample size and were beyond the scope of the current analysis. In addition, multiple comparisons were not corrected for, which may have increased the risk of false-positive findings. Therefore, these results should be considered hypothesis-generating and require validation in larger cohorts using appropriate statistical approaches to assess biomarker trajectories and account for potential confounders. Furthermore, although an increase in syndecan-1 concentrations over the two weeks following aSAH ictus tended to be more pronounced among patients who developed DCI than those who did not, it remains unclear whether glycocalyx breakdown is directly linked to DCI development. Additionally, several syndecan-1 and hyaluronan concentrations fell outside the detectable range of the ELISA standard curve, with concentrations being either below or above the detectable range. This limited the number of syndecan-1 and hyaluronan samples which could be used in the final analysis. The ELISA assay did not allow discrimination between measurements below the lower or above the upper detection limit; therefore, sensitivity analyses for censored values were not feasible. Another limitation is the potential for informative missingness and exclusion bias, as some patients did not have samples for the final measurement due to early mortality or due to the sample not being collected (Table S1). A formal missing-data modeling was not feasible due to the small sample size; however, we performed a descriptive assessment of excluded patients (Table S2). The excluded patients did not appear to differ substantially in key baseline characteristics. Moreover, patients who died before the final measurement were comparable to the overall cohort (syndecan-1: 27.76 ng/mL (IQR 25.41–33.24), hyaluronan 167.40 ng/mL (IQR 114.84–214.11)). Lastly, the diagnosis of DCI was established based on its clinical definition, which may lead to an underestimation of the incidence of DCI as silent ischemic lesions were not included.

Our study also has some strengths. Firstly, the simultaneous assessment of distinct glycocalyx components provided a more comprehensive understanding of glycocalyx dynamics after aSAH, offering novel insights into glycocalyx disruption following aSAH. Secondly, the prospective nature of this study with set time points at which plasma samples were collected allows for structured analyses and comparisons. The additional inclusion of a plasma sample within 24 h after DCI onset also enabled us to more specifically assess changes in glycocalyx components in relation to DCI onset.

### 4.5. Future Directions

Future research should further define the role of glycocalyx breakdown in aSAH and in the development of DCI in a larger cohort. Particularly, it should be established how specific glycocalyx components, including syndecan-1 and hyaluronan, are differentially shed in response to the different phases of aSAH and the processes leading to DCI development. If our findings are validated in larger cohorts, serial glycocalyx measurements may contribute to multimodal risk stratification for the development of DCI.

## 5. Conclusions

This study demonstrates that syndecan-1 concentrations increase during the first two weeks following aSAH ictus. This rise appeared to be more pronounced in patients that developed DCI, with a marked increase observed at DCI onset; however, these findings should be interpreted cautiously given the limited number of DCI patients. Hyaluronan concentrations were specifically high in the acute phase of aSAH as compared with concentrations in healthy individuals reported in the literature. Our findings suggest involvement of glycocalyx breakdown in the pathophysiology of DCI. Further research is warranted to explore the clinical potential of plasma glycocalyx biomarkers to predict DCI and improve our understanding of the potential role of the glycocalyx in the development of DCI.

## Figures and Tables

**Table 1:** Patient demographics.

Variable	Total (*N* = 29)	Non-DCI (*N* = 23)	DCI (*N* = 6)	*p*-Value
Mean age (SD)	58 (11.1)	59 (11.4)	52 (7.3)	0.269
Female, N (%)	24 (82.2%)	19 (81.9%)	5 (83.3%)	1.000
Smoking, N (%)	18 (61.5%)	13 (55.8%)	5 (83.3%)	0.362
Hypertension, N (%)	7 (24.1%)	4 (17.4%)	3 (50.0%)	0.131
WFNS, N (%) 1 2 3 4 5	17(58.6%) 4(13.8%) 1(3.4%) 2.(6.9%) 5 (17.2%)	13 (56.5%) 4 (17.4%) 1 (4.3%) 2 (8.7%) 3 (13%)	4 (66.7%) 0 0 0 2 (33.3%)	1.000
mFisher, N (%) 1 2 3 4	6 (20.7%) 0 13 (44.8%) 10(34.5%)	6 (26.1%) 0 9 (39.1%) 8 (34.8%)	0 0 4 (66.7%) 2 (33.3%)	0.113
Mortality, N (%)	4 (13.8%)	2 (8.7%)	2 (33%)	0.180

*N* = sample size, WFNS = World Federation of Neurosurgical Societies, mFisher = Modified Fisher Scale. Fisher’s exact test was used for dichotomous variables such as sex, smoking, hypertension and mortality. The Mann–Whitney U test was used for WFNS and mFisher, whereas the independent samples *t*-test was used for the mean age.

## Data Availability

The original contributions presented in this study are included in the article. Further inquiries can be directed to the corresponding author(s).

## Supplementary Materials

The following supporting information can be downloaded at: https://www.mdpi.com/article/10.3390/brainsci16080840/s1, Table S1: Overview of omitted samples, including total number of excluded measurements, distribution across non-DCI and DCI patients, and reasons for exclusion. Table S2: Characteristics of excluded measurements.
