# Supplementary material for: Plasma Biomarkers of the Glycocalyx in Delayed Cerebral Ischemia After Aneurysmal Subarachnoid Hemorrhage
Source: Brain Sci. 2026 Aug 7;16(8):840. doi: 10.3390/brainsci16080840 (PMC13510835; doi:10.3390/brainsci16080840)
Supplement: Supplementary file 1 [file brainsci-16-00840-s001.zip › brainsci-4443275-supplementary.pdf]

Supplementary Materials

Table S1: Overview of omitted samples, including total number of excluded measurements, distribution across non-DCI and DCI patients, and reasons for exclusion

**Biomarker**	**Timepoint**	**Total excluded samples (n)**	**Non-DCI patients excluded (n)**	**DCI patients excluded (n)**	**Reason for exclusion**
Syndecan-1	Baseline	1	1	0	• Outside assay range
Syndecan-1	Final measurement	8	6	2	• Outside assay range<br>• Patient died before sampling<br>• Sample not collected
Hyaluronan	Baseline	2	2	0	• Outside assay range
Hyaluronan	Final measurement	9	7	2	• Outside assay range<br>• Patient died before sampling<br>• Sample not collected

Table S2: Characteristics of excluded measurements

Variable	Patients with missing samples (N=9)
Mean age (SD)	58 (13.8)
Hypertension, N (%)	2 (22.2)
WFNS, N (%)	
- 1	- 6 (66.7)
- 2	- 0
- 3	- 1 (11.1)
- 4	- 2 (22.2)
- 5	- 0
mFisher, N (%)	
- 1	- 3 (33.3)
- 2	- 3 (33.3)
- 3	- 3 (33.3)
- 4	- 0

N = sample size of patients with missing data, WFNS = World Federation of Neurosurgical Societies, mFisher = Modified Fisher Scale. Age, hypertension status, WFNS grade, and mFisher grade of patients with missing samples appeared to be comparable to those of the overall cohort, which suggests that the exclusion of samples was unlikely to have substantially affected our results.
